# Novel Titin Gene Mutation Causing Autosomal Dominant Limb-Girdle Muscular Dystrophy

**DOI:** 10.7759/cureus.30550

**Published:** 2022-10-21

**Authors:** Leema Reddy Peddareddygari, Kinsi Oberoi, Raji P Grewal

**Affiliations:** 1 Research and Development, Dynamic Biologics Inc., Monmouth Junction, USA; 2 Life Sciences Division, Clarivate Analytics, Philadelphia, USA; 3 Neuroscience Institute, Saint Francis Medical Center, Trenton, USA

**Keywords:** novel mutation, titin gene, contractures, limb girdle muscular dystrophy, titinopathy

## Abstract

We report a genotype-phenotype analysis of a family in which a titinopathy is transmitted in an autosomal dominant pattern. In this family, following neurological history and examination, electromyogram, and muscle biopsy, the diagnosis of limb-girdle muscular dystrophy with contractures was made in an affected mother and son. Genetic testing employing the whole exome was performed and revealed two variants in the *TTN* gene, c.712G>C, p. Glu238Gln and c.1397A>C, p.Gln466Arg, which segregated with the disease in the affected mother-son duo but not in an unaffected sibling. Although protein modeling suggests that the c.712G>C, p. Glu238Gln polymorphism is damaging, it has been reported in the Genome Aggregation Database which includes exome and genome sequence data of unrelated individuals sequenced as part of various disease-specific and population genetic studies. In contrast, the c.1397A>C, p.Gln466Arg variant is novel and has not been reported in any public genetic databases or our internal laboratory database. Protein modeling analysis indicates that p.Gln466Arg is damaging and we hypothesize that it is the disease-producing mutation resulting in muscular dystrophy. Our research report expands the spectrum of mutations causing titinopathy.

## Introduction

The *titin *gene, *TTN*, encodes a large multifunctional polypeptide that participates in myogenesis and contributes to the elasticity of muscle tissues [1]. Mutations in this gene result in a variety of clinical and pathological phenotypes ranging from early-onset myopathy with fatal cardiomyopathy and congenital centronuclear myopathy to adult-onset muscular dystrophy [2]. The pattern of inheritance for titinopathies can vary from autosomal recessive to autosomal dominant. We present a family carrying a novel mutation in the *TTN *gene associated with a limb-girdle phenotype and transmitted in an autosomal dominant pattern of inheritance.

## Case presentation

Clinical presentation

At age 33 years, the index patient (II-1) was evaluated for a minor head injury and had a normal neurological examination. Then, at age 42 years, she was referred for neurological consultation with complaints of weakness in her arms and legs unassociated with numbness, double vision, ptosis, or dysphagia. The onset of the weakness developed over several years with difficulty climbing stairs, arising from a low-seated chair and using her arms above her head. Neurological evaluation revealed a normal mental status, cranial nerve examination, and tests of sensation. She had proximal muscle weakness Medical Research Council (MRC) Grade 4/5 and was diagnosed with a myopathy. Serum chemistries revealed elevated creatine phosphokinase (CPK), lactate dehydrogenase, and serum glutamic pyruvic transaminase consistent with a myopathic process (Table 1).

**Table 1:** Laboratory investigation results for the index patient (II-1)

Investigations	Results	Units	Reference range
Creatine phosphokinase	1731	MU/mL	0-54
Lactic dehydrogenase	638	IU/L	110-225
Serum glutamic pyruvic transaminase	71	IU/L	0-50

An electromyogram showed normal motor and sensory nerve conduction parameters and myopathic changes on the needle examination of the proximal muscles with minimal inflammatory features. A muscle biopsy of the deltoid was performed and interpreted as showing evidence of a combination of neurogenic and myopathic features with minimal inflammation. She was treated with high-dose prednisone without a significant response. Over the next 23 years, she sought multiple neurological consultations and with no clear diagnosis or response to any treatment for presumed inflammatory myopathy. She continued to progress without the involvement of the cranial nerves. At age 65 years, her neurological examination showed a normal mental status and sensory and cranial nerve examination. She was diffusely weak in her arms and evaluation of the power of the biceps, deltoid, supraspinatus, and infraspinatus muscles showed MRC 4/5 weakness. Assessing her legs showed Grade 1/5 testing hip flexion, hip adduction, abduction with some increase in power in testing knee flexion and extension Grade 2/5 and 4/5 distally (foot dorsiflexion, eversion, and inversion). She had contractures in assessing mobility at the knees, ankles, and shoulder. She could not stand or walk independently and was diffusely hyporeflexic with flexor plantar responses bilaterally. She had no abnormalities of the cerebellar system.

**Figure 1:**
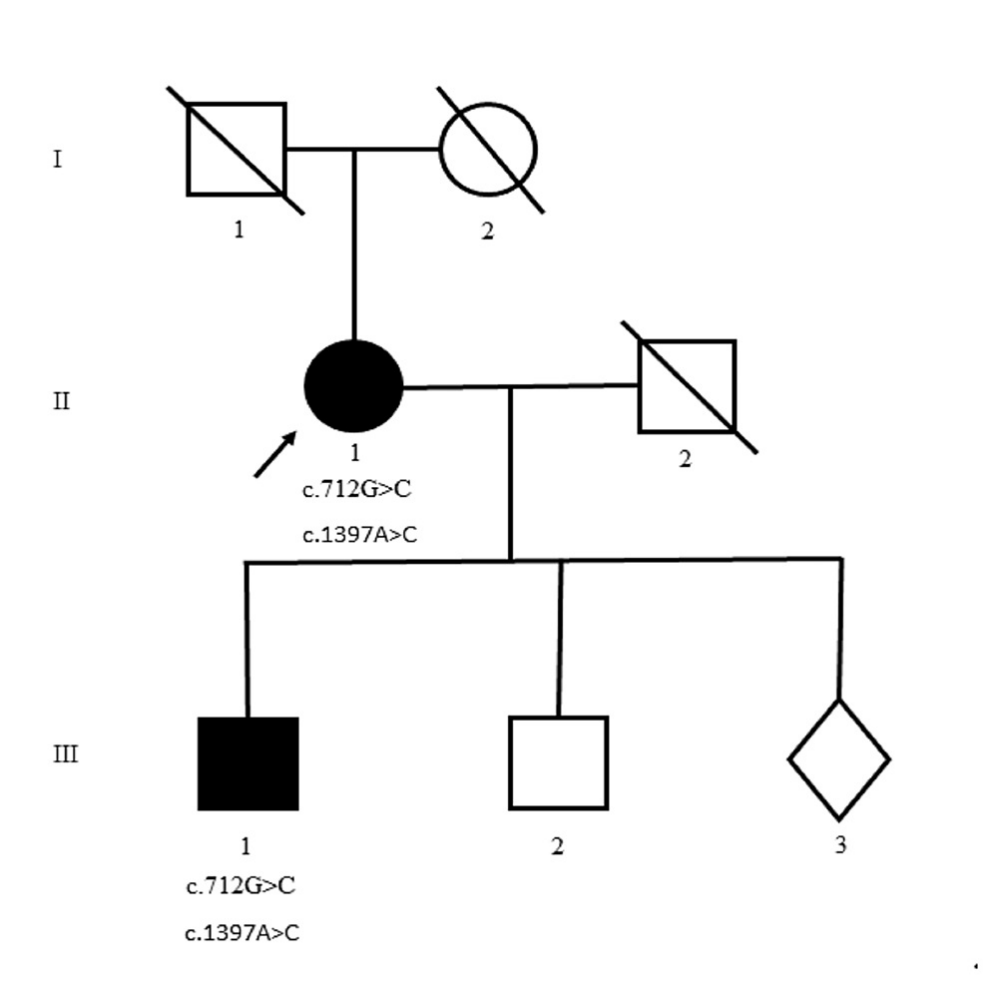
Pedigree depicting the affected mother and son with a limb-girdle muscular dystrophy with contractures phenotype inheriting the same TTN variants. Different generations are represented by the roman numerals. Squares represent males, circles represent females, and diamond indicates gender unknown. Affected individuals are represented by dark fill. Arrow indicates the index patient.

Family history revealed that both of her parents were deceased and had lived beyond their 80s and did not suffer from any neurological condition. In addition, she had one sister and six brothers, and none of them were affected by a neurological disorder. She has three children, one of whom, a son (III-1), was affected by a neuromuscular condition (Figure 1).

Her son, III-1, was able to run as a teenager and during childhood did not walk on his toes. In his early twenties, he had difficulty in climbing stairs and getting out of the chair. In his later twenties, he started to have difficulty using his arms. He had no complaints of numbness, visual symptoms, chewing, or swallowing. At age 27 years, he presented for a neurological examination and displayed no abnormalities of his mental status or cranial nerve examination. The tests of sensation were normal and he had diffusely reduced reflexes with flexor plantar responses. He had MRC Grade 4/5 weakness of the proximal muscles of his arms and legs. In addition, contractures were noted in assessing neck flexion, and range of movement at the elbows and ankles. Laboratory testing showed elevated CPK levels of 6,319 IU/L (reference range 24-204). A muscle biopsy was performed demonstrating the presence of a myopathic process most consistent with muscular dystrophy. Over the next decade, serial neurological examinations demonstrated a progressive decline in power and increasing development of more contractures. Neurological examination at age 40 years was similar to that performed earlier except that he now was areflexic and had increased weakness of the proximal arms and legs in the MRC Grade 2-3/5 range. Since the presence of contractures could indicate Emery Dreifuss muscular dystrophy (EMD), a cardiac evaluation was performed and showed a normal ejection fraction. An evaluation by a cardiologist revealed no significant cardiac abnormalities.

The muscle biopsies of both patients were retrieved and re-analyzed. The muscle biopsy of the index patient (II-1) showed the presence of fiber splitting, internal nuclei, and mild inflammation. In addition, a rare inclusion body was noted but, overall, the evidence was more suggestive of a myopathic process than a neurogenic process. Analysis of the muscle biopsy of individual III-1 showed similar features with minimal inflammation and some inclusion bodies were noted. In addition, staining for emerin and collagen V1 proteins was negative. The stains for collagen V1 were performed to investigate Ullrich's congenital muscular dystrophy known to be associated with contractures.

An evaluation of an unaffected brother (III-2) at age 32 years revealed no history of weakness and a normal neurological examination.

Genetic testing

During the course of his care, individual (III-1) had undergone the most current genetic testing that was available commercially for muscular dystrophies. These included tests for the genes causing dystrophinopathies and EMD, which were negative. After presenting for evaluation to our institution, blood samples were obtained from the index patient (II-1) and two of her children (III-1, III-2) following an informed consent process approved by IRB. Genomic DNA was extracted and whole-exome sequencing of the index patient was performed. The nucleotide-level variation analysis of the exome sequence data was analyzed using the DNA nexus platform. Paired-end reads were mapped to the reference human genomes (GCF_000001405.13, UCSC NCBI37/hg19) using the Burrows-Wheeler Alignment tool. Variants were called by the GATK-lite variant caller and the variant file (VCF) was annotated using the VEP (Variant Effect Predictor) app on Ensambl.org. The annotated file obtained from VEP was further filtered using in-house scripts to identify variants in 134 known myopathy genes. The variants were further analyzed to filter out common variants as well as variants present in our internal database at the Neurogenetics Foundation. The internal database is a collection of whole-exome sequencing data of 62 patients with a neurological history that included rare gene variants with a frequency of less than 3% along with their potential pathogenicity data generated using SIFT [3], Polyphen [4], and Mutation Taster [5] protein analysis tools. In this case, we further filtered for rare variants with minor allele frequency (MAF) <1% and single-nucleotide polymorphisms (SNPs) predicted as damaging by all three prediction software tools.

We filtered variants in 85 genes established to cause muscular dystrophies (https://neuromuscular.wustl.edu/musdist/lg.html) including genes known to cause EMD, *Emerin*, *Lamin A/C*, *SYNE1*, *SYNE2*, *FHL1*, *TMEM43*, *Titin,* and EMD modifiers *SUN1* and *SUN2* genes.

We identified one heterozygous variant in the *CAPN3* gene, rs147764579, NM_000070.3(CAPN3):c.1466G>A (p.Arg489Gln), which has been reported as pathogenic in the ClinVar database (https://www.ncbi.nlm.nih.gov/clinvar/variation/289644/). We identified two variants in *SYNE1* gene (EDMD4), rs150589796, NM_182961.4 (*SYNE1*):c.23002C>G (p.Leu7668Val) and rs114954026, NM_182961.4 (*SYNE1*):c.9764C>T (p.Ser3255Leu), and both heterozygous missense mutations were predicted to be benign/likely benign in ClinVar database. 

**Figure 2:**
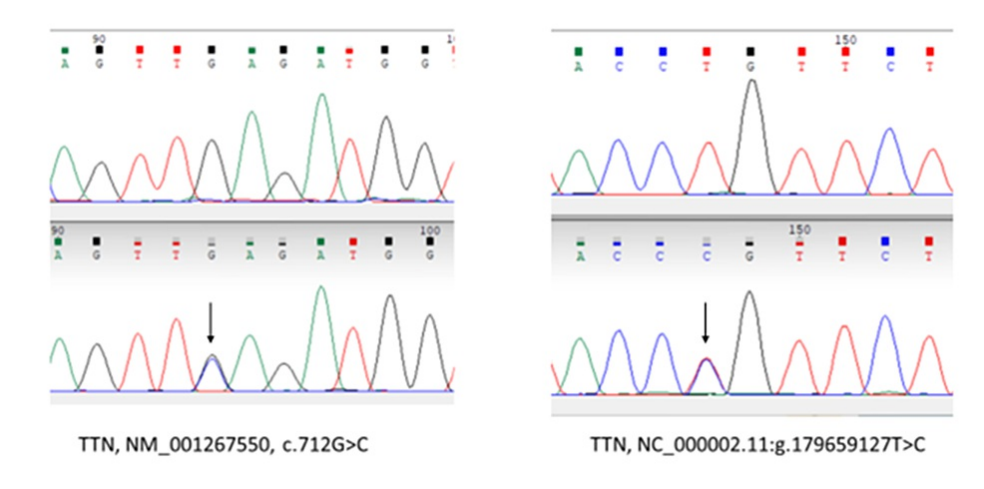
Chromatograph showing the wild (top) and the mutant (bottom indicated by arrow) peaks in TTN gene identified in the unaffected son and the index patient.

We also identified two variants in the *TTN* gene (Figure 2).

A novel heterozygous variant in exon 6, Z-disk (N-terminus) of *TTN*, NM_001267550, c.712G>C, p. Glu238Gln, is not reported in ClinVar nor found in 1000G or Genome Aggregation Database (GnomAD) databases and predicted to be a disease-causing polymorphism by Mutation Taster, unknown by SIFT, and probably damaging by PolyPhen.

The second variant in exon 8, Z-disk of *TTN,* NC_000002.11:g.179659127T>C NM_001267550.2:c.1397A>C,p.Gln466Arg, rs150282120, is not reported in ClinVar but is listed in the SNP database (https://www.ncbi.nlm.nih.gov/snp/rs150282120#hgvs_tab) and reported in GnomAD exome database (het 6/251336) (https://gnomad.broadinstitute.org/variant/2-179659127-T-C?dataset=gnomad_r2_1). It is predicted as deleterious by SIFT, unknown by PolyPhen analysis, and disease-causing polymorphism by Mutation Taster. The Mutation Taster tool also predicted that the splice site is affected by the loss of the Z-repeat 2 region. This in turn may affect gene function downstream from this altered splice site. To investigate splice site effects, this variant was further analyzed using Human Splicing Finder (HSF) [6], Alternative Splice Site Predictor (ASSP) [7], and EX-SKIP [8] tools to determine if it can interfere with normal splicing. The HSF tool predicted alteration in the exonic ESE site with potential alteration of splicing, the ASSP tool predicted the variant results in a decrease in the splice site strength, and EX-SKIP predicted that the mutant allele sequence had a higher chance of exon skipping than the wild allele sequence. All of these modeling programs suggest that the splice site is likely to be affected by this variant.

The variants in *CAPN3*, *SYNE1,* and *TTN* genes were further confirmed by Sanger sequencing in the index patient and two sons, one affected and the other unaffected (Table 2).

**Table 2:** Sanger sequencing results for variants identified by whole-exome sequencing of the index patient (II-1). SNP, single-nucleotide polymorphism.

SNP (gene)	Mother II-1 (affected)	Son III-1 (affected)	Son III-2 (unaffected)
rs147764579 (*CAPN3*)	G/A	G/G	G/A
rs114954026 (*SYNE1*)	G/A	G/G	G/G
rs150589796 (*SYNE1*)	G/C	G/G	G/G
rs150282120 (*TTN*)	A/C	A/C	A/A
Exon 6, c.712G>C (*TTN)*	G/C	G/C	G/G

## Discussion

The index patient was initially considered to have polymyositis following muscle biopsy. A genetic myopathy became a diagnostic consideration after she failed to respond to treatment and one of her sons became symptomatic. Whole-exome sequencing identified a pathogenic variant in the*CAPN3* gene, rs147764579, and NM_000070.3 (*CAPN3*):c.1466G>A (p.Arg489Gln) (Table 2). This variant is transmitted from the index patient to her unaffected son and not to the affected son, indicating that this is not the pathogenic mutation in this family. In addition, the *SYNE1* variants detected are found only in the mother and not in the affected son excluding them as candidate mutations causing the myopathy.

Sanger sequencing confirmed the presence of the two variants in* the TTN* gene in this family, NM_001267550, c.712G>C, p. Glu238Gln and NM_001267550.2:c.1397A>C, p.Gln466Arg (Table 2). Interestingly, both variants were present in the affected mother and son and not in the unaffected son. Our analysis demonstrates that these two *TTN* variants are in cis. The c.712G>C, p. Glu238Gln variant is not found in the publicly available databases and represents a novel mutation. Our protein modeling analysis suggests that this is a disease-producing variant. Interestingly, another variant at this site, rs867879786, NM_001267550.2:c.712G>A, p.Glu238Lys, has been reported in dbSNP; however, there is no frequency reported and it is not reported in the ClinVar database.

The second *TTN* variant detected in this family, NM_001267550.2, c.1397A>C, p.Gln466Arg, is rare and in the GnomAD database has a frequency of 6/251,336, and our variant analysis suggests that it likely causes a splicing error. The GnomAD databases consist of control subjects in studies of adult-onset diseases such as psychiatric disorders, type 2 diabetes mellitus, and cardiovascular disease [9]. It is highly unlikely that six individuals with a myopathy would participate as controls in these studies. This indicates that this mutation alone is not sufficient to result in muscular dystrophy.

Following the ACMG/AMP guidelines although both the *TTN* variants in this family are pathogenic (or probably pathogenic), we hypothesize that the novel c.712G>C, p. Glu238Gln is disease producing in this family [10]. This hypothesis is supported by our protein modeling results indicating that this mutation results in the pathogenicity of the resultant protein. In addition, the absence of this variant in large databases with healthy control populations confirms that this is an extremely rare mutation. There may be a potential pathogenic role of the second variant, c.1397A>C, p.Gln466Arg, in this family. Since this variant has been reported in neurologically normal individuals, perhaps it is a modifier of the effect of the novel c.712G>C, p. Glu238Gln mutation. There is a precedent for double mutations occurring in cis resulting in a given phenotype [11,12-14]. It is possible that there is a combined effect of the two variants on the protein both located in N-terminus which is embedded in the Z-disk that acts as a mechano-sensor that results in muscular dystrophy [15]. This issue will be further clarified by reports of additional families carrying the c.712G>C, p. Glu238Gln mutation alone.

There are several other interesting features involving both the genotype and phenotype in this family. First, there is a lack of family history of disease in the parents of the index patient. Since this is an autosomal dominant condition, there are two possibilities, a spontaneous mutation or non-paternity. We suspect the p. Glu238Gln variant represents a spontaneous germline mutation. The phenotype of the mother-son duo is consistent with a limb-girdle phenotype with contractures with a difference in age of onset. The onset of symptoms prompting medical attention was about age 40 years in the mother compared with the onset at 20 years in the affected son. Autosomal recessive limb-girdle muscular dystrophy-10 (LGMD-10) caused by *TTN* mutations has been reported previously [16,17]. Unlike the published cases, the disease in this family is transmitted in an autosomal dominant pattern of inheritance. There was some consideration of an EMD in this family. The EMD phenotype caused by *TTN* mutations has been previously reported in three families of French and Algerian descent. In these families, it follows an autosomal recessive pattern of inheritance, with disease onset at 3-10 years of age with symmetric limb-girdle weakness progressing such that the patients became wheelchair-bound by 13-36 years of age. The clinical features in these patients included contractures and decreased respiratory capacity but without any cardiac involvement [18]. In our family, there was no evidence of either cardiac or respiratory involvement. However, contractures are not a feature exclusive to EMD and has been observed in many other genetic myopathies [19].

Our analysis does not provide any genetic data that could account for the intrafamilial variability between the mother and her son both in terms of age of onset and the phenotype. The polymorphisms observed in the *CAPN3* and *SYNE1* do not show relevant differences between the affected pair of mother and son and exclude them as genetic modifiers. It is possible that the intrafamilial variability is related to sex with a milder phenotype in the mother.

## Conclusions

In this study, we report a novel mutation, c.712G>C, p. Glu238Gln, that can result in a limb-girdle phenotype with contractures and follows an autosomal dominant pattern of inheritance. Our study expands the phenotypic heterogeneity of titinopathies and the mutational spectrum in the *TTN* gene. It further demonstrates the power of next-generation sequencing and how it can facilitate patients with undiagnosed genetic disorders.
